# Advancing Health Behavior Theories in Research and Practice

**DOI:** 10.3390/ejihpe16040051

**Published:** 2026-04-01

**Authors:** Yifei Liu, Dhananjay Nayakankuppam

**Affiliations:** 1 Division of Pharmacy Practice and Administration, University of Missouri-Kansas City School of Pharmacy, Kansas City, MO 64108, USA; 2 Department of Marketing, Tippie College of Business, University of Iowa, Iowa City, IA 52242, USA

## 1. Overview of the Topic

Understanding health behaviors is fundamental to designing effective health interventions. Health behavior theories provide structured frameworks for explaining why individuals engage in specific health-related actions and how these actions may change over time. These theoretical perspectives guide not only the conceptual understanding of behavior but also the methodological design of research and interventions aimed at promoting better health outcomes. Over the past several decades, a range of theoretical frameworks has contributed to the development of behavior science, and these frameworks have informed numerous interventions across diverse contexts (Glanz et al., 2015). However, contemporary healthcare systems face complex challenges. Aging population, rising prevalence of chronic diseases, technological transformations in healthcare delivery, and health inequities have highlighted the need for continued refinement of health behavior theories. Behavioral research must therefore evolve to integrate interdisciplinary perspectives and generate insights that can translate effectively into clinical practice. This Topic brings together contributions that collectively aim to advance the understanding and application of health behavior theories. The intended audience for this Topic includes researchers, healthcare practitioners, and policymakers working at regional, national, and international levels.

## 2. Contributions to Literature

This Topic represents a collaborative effort across five participating journals to expand knowledge on the development, refinement, and application of health behavior theories. The collection comprises twelve empirical studies (including one study with a correction) and one systematic review, all grounded in theoretical frameworks and examining a range of health behaviors. The scope of the Topic encompasses multiple areas of health behavior research. One area involves the testing and refinement of theoretical models. As health contexts change and new evidence emerges, it becomes essential to evaluate existing frameworks and explore modifications or extensions that better capture the complexity of behavior change processes. Another area concerns the identification of predictors and determinants of health behaviors. By examining cognitive, social, and environmental influences on behavior, research can provide insights that guide the development of more effective interventions. Measurement and methodological development also play a role in advancing behavioral science. Valid and reliable measures of theoretical constructs are essential for testing models and evaluating interventions. In addition, the Topic emphasizes the translation of theoretical knowledge into practical applications. Health behavior theories are most valuable when they inform the design and implementation of interventions that improve health outcomes. Research addressing intervention development, health promotion strategies, and evaluation of healthcare services demonstrates how theoretical insights can be translated into practice (Glanz et al., 2015). The Topic also reflects the diversity of healthcare settings in which behavioral research is conducted. Studies span a wide range of contexts, which underscores the importance of applying behavioral theories across different levels of healthcare delivery and across patient populations.

## 3. Remaining Challenges

Despite the advances demonstrated in this Topic, several challenges remain in the study and application of health behavior theories. One challenge is the integration of individual-level theories with broader perspectives. Health behaviors are influenced not only by individual cognition and motivation but also by social networks, institutional structures, and policy environments. Expanding theoretical frameworks to incorporate these multi-level influences remains an important area for future research. Measurement challenges also persist. Accurately assessing psychological constructs such as attitudes and self-efficacy can be difficult, particularly when relying on self-reported data. Advances in digital technologies and data collection methods may offer opportunities to enhance measurement precision and capture behavioral processes in real time. Finally, translating theoretical insights into sustainable health interventions continues to be a complex task. Bridging the gap between theory and practice requires collaboration between researchers, practitioners, and policymakers to ensure that theoretical models inform practical decision-making in healthcare systems.

Looking ahead, a few directions for future research emerge from the themes of this Topic. First, interdisciplinary collaboration will remain essential for advancing behavioral science. Integrating perspectives from public health, behavioral psychology, sociology, and health services research can contribute to more comprehensive models that reflect the multifaceted nature of health behaviors. Second, methodological innovation should continue to be a priority. Emerging tools such as digital health platforms, wearable technologies, and real-time behavioral monitoring systems may provide new opportunities to study behavior dynamically and evaluate interventions more effectively.

## 4. Conclusions

The articles included in this Topic highlight the enduring importance of health behavior theories for understanding and improving health outcomes. By exploring theoretical constructs, examining behavioral determinants, and translating insights into practical applications, the Topic aims to advance health behavior research. At the same time, the collection underscores the need for continued theoretical refinement, methodological innovation, and interdisciplinary collaboration. Addressing these areas will help ensure that health behavior theories remain responsive to evolving healthcare challenges and capable of guiding effective interventions that promote health outcomes across patient populations.
